# Prevalence of Intestinal Parasite Infection and Their Associated Factors Among University Students and University Food Handlers in Ethiopia: A Systematic Review and Meta-Analysis

**DOI:** 10.1155/japr/9522730

**Published:** 2025-09-30

**Authors:** Getu Abeje, Ayenew Berhan, Ayenew Assefa, Andargachew Almaw, Mitikie Wondmagegn, Mulat Erkihun, Dessie Tegegne, Bekele Sharew, Teklehaimanot Kiros, Getaneh Tegegne, Molla Getie, Habtu Debash, Gebrie Kassaw Yirga, Anteneh Mengist Dessie

**Affiliations:** ^1^Department of Medical Laboratory Science, College of Health Science, Debre Tabor University, Debre Tabor, Ethiopia; ^2^Department of Medical Laboratory Science, College of Medical and Health Science, Injibara University, Injibara, Ethiopia; ^3^Department of Medical Laboratory Science, College of Medical and Health Science, Wollo University, Dessie, Ethiopia; ^4^Department of Nursing, College of Health Science, Debre Tabor University, Debre Tabor, Ethiopia; ^5^Department of Public Health, College of Health Science, Debre Tabor University, Debre Tabor, Ethiopia; ^6^Flinders Health and Medical Research Institute, Flinders University, Bedford Park, Adelaide, Australia

## Abstract

Intestinal parasite infections remain a significant public health concern in developing countries. Food handlers are particularly vulnerable to various enteric pathogens, which can then spread to the wider community. There is an urgent need for a comprehensive national review to consolidate findings from existing studies and draw conclusive insights. This systematic review and meta-analysis is aimed at determining the overall prevalence of intestinal parasites and evaluating the pooled odds ratios related to hygiene practices among university students and food handlers in Ethiopia. The review included articles from multiple online peer-reviewed databases such as PubMed, Medline, Google Scholar, and CINAHL, along with unpublished gray literature containing full-text studies in English. A random-effects model with a 95% confidence interval (CI) was utilized, employing STATA Version 17 statistical software for analysis. Forest plots were generated to calculate the prevalence of intestinal parasites, while the *I*^2^ heterogeneity test was used to assess variability among the studies. Additionally, subgroup and sensitivity analyses were performed, and funnel plots along with Egger's regression tests were conducted to evaluate potential publication bias. This systematic review encompassed 101 studies to estimate the prevalence of intestinal parasitic infections among food handlers and university students in Ethiopia. Of these, 15 studies met the inclusion criteria for analysis. The findings indicated that approximately 29.65% of food handlers were infected with intestinal parasites (95% CI: 22.79%–36.5%). The most frequently identified parasites included *Entamoeba histolytica* (11.52%), *Ascaris lumbricoides* (9.34%), and *Giardia lamblia* (5.85%). Significant risk factors associated with intestinal parasitic infections included having untrimmed fingernails (AOR = 3.58; 95% CI: 2.49, 5.16) and the inability to wash hands (AOR = 3.86; 95% CI: 1.97, 7.58). Therefore, emphasis on routine parasite screenings and educational initiatives targeting proper nail trimming and hand hygiene practices is essential to prevent foodborne illnesses and protect the health of vulnerable populations as well as highlighting the interplay of geography, study design, detection methods, and personal hygiene in the broader effort to reduce prevalence and enhance public health outcomes.

## 1. Introduction

Foodborne illnesses pose a significant global public health challenge and are receiving increasing attention [1]. According to the World Health Organization, there are approximately 600 million cases and around 420,000 deaths attributed to these illnesses each year worldwide [2]. This problem is particularly severe in developing countries, where poor hygiene practices during food preparation and handling lead to higher prevalence rates [3]. Intestinal parasitic infections (IPIs) are among the leading contributors to foodborne diseases [4, 5]. Each year, more than 2 billion cases and 200,000 deaths are reported globally [6]. These diseases can spread through the consumption of contaminated food or water, as well as through person-to-person contact via fecal–oral transmission [7]. The global prevalence of IPIs varies depending on the specific type of parasite. *Ascaris lumbricoides*, *Trichuris trichiura*, and hookworms are the most common helminthic parasites, while *Giardia lamblia* and *Entamoeba histolytica* are the most prevalent protozoan infections. Helminth infections, in general, are more widespread [6, 8, 9].

In addition to causing illness and deaths, intestinal parasites can negatively impact psychological and social well-being by inducing nutritional deficiencies, resulting in stunted growth, low levels of vitamin A, iron deficiency anemia, and weight loss [10]. Numerous infectious diseases are transmitted through contaminated food and water, and intestinal parasites are a significant cause of foodborne illnesses among food handlers [11].

Despite continued efforts to combat IPIs, they remain a significant cause of illness and mortality worldwide [12, 13], particularly in Sub-Saharan Africa [11], including Ethiopia. Various factors contribute to the high prevalence of these infections, such as low socioeconomic status, limited access to clean water and proper sanitation, inadequate healthcare services, low public awareness, and unfavorable environmental and climatic conditions [9, 14]. In Ethiopia, the most common intestinal protozoan and helminthic parasites include *G. lamblia*, *Entamoeba histolytica/dispar*, and *A. lumbricoides* [15].

Research conducted in Ethiopia has shown significant variations in the prevalence rates of intestinal parasite infections among food handlers in various public food establishments. For example, student cafeterias at Haramaya University reported a prevalence rate of 14.3% [16]. In contrast, Gojjam Prison exhibited a much higher prevalence of 61.9% [17]. Other findings include prevalence rates of 15% at Wollo University cafeterias [18], 14.5% in Aksum City [19], 33% at Jimma University Specialized Hospital [20], 45.3% at the Students' Cafeteria at Addis Ababa University [11], 52.4% at Mekelle University students' cafeteria [3], and 20.6% at Hawassa University [21].

Furthermore, in low-income countries like Ethiopia, food handlers are often hired without proper screening for infectious diseases, a practice closely tied to inadequate hygiene habits [12, 22]. To safeguard the health of students who consume food and drinks in significant quantities, maintaining high standards of hygiene in food handling and preparation is crucial. This is especially critical as public universities in Ethiopia have experienced rapid expansion in the last two decades, heightening the risk of foodborne illness outbreaks.

Previous research has shown a significant variance in the prevalence of IPIs among food service workers, particularly in university student cafeterias. However, the root causes of these diverse and unpredictable rates, as well as the hygienic factors influencing them, have not been thoroughly investigated in Ethiopia. While there has been a rise in published articles focusing on the epidemiology of intestinal parasites in recent years, the burden of these parasites continues to increase [14].

Therefore, research endeavors such as systematic reviews and meta-analyses are crucial for summarizing and critically evaluating existing studies to determine the overall prevalence, with a focus on community well-being and public health initiatives. To address this need, a systematic review and meta-analysis were conducted in Ethiopia to establish the pooled prevalence of intestinal parasites and associated factors among food handlers employed in university settings.

## 2. Methods

### 2.1. Reporting and Protocol Registration

**Figure 1:**
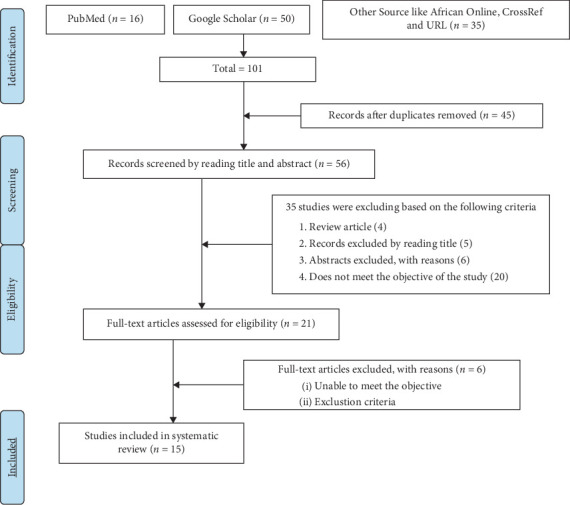
PRISMA flow diagram depicting the study selection process for the pooled prevalence of intestinal parasitic infections among food handlers in Ethiopia, 2024.

The systematic review and meta-analysis is aimed at determining the overall prevalence and associated factors of IPIs among university students and food handlers in university settings in Ethiopia. As of 2020, Ethiopia's population was approximately 113,881,451, with around 21.3% living in urban areas, according to the referenced document. The findings of the study were reported following the Preferred Reporting Items for Systematic Reviews and Meta-Analyses (PRISMA) guidelines, which detail the search and selection process of the included studies [23] (Figure 1). Additionally, the protocol for this systematic review and meta-analysis has been registered with the International Prospective Register of Systematic Reviews (PROSPERO) under the reference number CRD42024497063. For further information, you can access the registration via CRD-register@york.ac.uk or visit the website at https://www.york.ac.uk/inst/crd.

### 2.2. Search Strategy

English articles were sought from a variety of international databases, including PubMed/Medline, Google Scholar, African Online, and CrossRef. Additionally, gray literature was examined through reference reviews, and relevant unpublished papers were sourced from online repositories, including the libraries of Addis Ababa University, Mekelle University, Jimma University, Haramaya University, Hawassa University, and the University of Gondar.

The literature search was carried out from April 10 to May 25, 2024, using Medical Subject Headings (MeSH) terms and entry terms such as “prevalence,” “intestinal parasite,” “disease,” “food processing,” “associated factors,” “food handlers,” “university student cafeterias,” “university students,” and “Ethiopia.” These terms were combined using Boolean operators “OR” and “AND.” MeSH terms and entry terms within the same category were searched with “OR,” while “AND” was employed to link different categories. The entry terms were adjusted as necessary to meet the specific requirements of each database. The studies retrieved from the databases were exported to EndNote Version 8 reference management software for organization and management [24].

### 2.3. Eligibility Criteria

#### 2.3.1. Inclusion Criteria

This review includes case–control, cohort, and cross-sectional study designs. The literature search encompassed both published and unpublished articles written in English. It specifically focused on articles that examined the prevalence of intestinal parasites and related variables among university students and food handlers. The review considers studies published from January 2014 to 2024 G.C.

#### 2.3.2. Exclusion Criteria

Articles that reported on the pooled prevalence of intestinal parasites among university students and food handlers but failed to provide specific data on prevalence or associated factors were excluded from this review. Additionally, studies for which the required information could not be obtained, even after contacting the corresponding authors, were also excluded. Furthermore, editorial letters, comments, case reports, case series, and monthly and annual police were also excluded.

### 2.4. Definition of IPI and Outcome Measures

IPI is defined as an infection caused by one or more parasites that may affect individuals engaged in cafeteria services, aiding in the implementation of effective preventive and control measures. To calculate the prevalence of IPIs, the total number of cases will be divided by the total number of food handlers involved in the study and then multiplied by 100. The association between IPIs and various related factors will be evaluated using the log odds ratio.

The main aim of the study is to determine the overall prevalence or proportion of intestinal parasites among university students and food handlers. The secondary objective is to pinpoint the factors linked to intestinal parasites in both university students and food handlers.

### 2.5. Data Extraction

After identifying eligible articles, two independent reviewers (GA and AB) extracted the relevant data using a structured format in a Microsoft Excel spreadsheet. In instances where discrepancies occurred in the extracted data, the extraction process was repeated. Any disagreements regarding the data were resolved through discussions with one of the authors (HD).

A data extraction template was developed based on the Joanna Briggs Institute (JBI) data extraction form for systematic reviews and research syntheses [25]. Prior to the actual data extraction, all members of the review team practiced the extraction process independently in Microsoft Excel. The data extraction tool included essential information such as the first author's name, year of publication, study design, study area, study period, region of study, population (participants), laboratory methods, species of intestinal parasites, prevalence of intestinal parasites, and total sample size. In cases where data were unclear, communication was established with the corresponding authors (GA) of the primary studies. Subsequently, the data were extracted using the Microsoft Excel spreadsheet, and the log odds ratio for each associated factor was calculated.

### 2.6. Quality and Risk of Bias Assessments

The Joanna Briggs Institute Meta-Analysis of Statistics Assessment and Review Instrument (JBI-MAStARI) was utilized to critically assess the quality of the studies included in the review. The quality appraisal considered several components, such as clear inclusion criteria, thorough descriptions of the study area and subjects, criteria for measuring both dependent and independent variables, appropriate statistical analyses, adequate sample sizes, and satisfactory response rates. Studies scoring ≥ 3 out of 5 were deemed to have good quality.

Two authors (AM and GA) independently evaluated the quality of each original study using the JBI quality assessment tool. Only studies that achieved a quality score of 50% or higher were included in the final systematic review. The assessment was based on the following questions: (1) Was the sample frame appropriate to address the target population? (2) Were study participants sampled in an appropriate manner? (3) Was the sample size adequate? (4) Were the study subjects and the setting described in detail? (5) Was the data analysis conducted with sufficient coverage of the identified sample? (6) Were valid methods used for the identification of the condition? (7) Was the condition measured in a standard, reliable way for all participants? (8) Was there appropriate statistical analysis? (9) Was the response rate adequate, and if not, was the low response rate managed appropriately? (Table 1). Any discrepancies that arose during the critical appraisal process were resolved through discussions between the two authors (MG and DT).

### 2.7. Data Synthesis and Statistical Analysis

The study was conducted among university students and food handlers working in universities. The extracted data were imported into STATA Version 17 software for analysis. A narrative description of the included studies was provided. A random-effects model was utilized to estimate the overall pooled prevalence or proportion of intestinal parasites and associated factors among university students and food handlers. Heterogeneity was evaluated using the *I*^2^ statistic and Cochran's *Q* statistic, with a significance level set at 5%. *I*^2^ coefficients of 25%, 50%, and 75% typically indicate low, moderate, and high heterogeneity, respectively. Significant heterogeneity is identified when the *p* value is below 0.05 and *I*^2^ is greater than 50%. To explore sources of heterogeneity or to minimize random variations among the primary study estimates, subgroup analysis and meta-regression were conducted based on regional levels, study areas (community or institution), and sample size characteristics. Additionally, sensitivity analysis was performed to assess the impact of individual studies on the overall estimates.

For studies with more than 10 entries, publication bias was evaluated using funnel plots and Egger's test, with a *p* value below 0.05 indicating potential publication bias. Effect size was used to analyze the relationship between associated factors and intestinal parasites among university students and food handlers, with a *p* value below 0.05 signifying statistical significance. The pooled point prevalence, along with 95% confidence intervals, was presented in a forest plot format. Following statistical calculations, the results indicated significant heterogeneity among the studies (*I*^2^ = 96.80%, *p* < 0.001). To determine the overall proportion of intestinal parasites, a back-transformation of the weighted mean of the transformed proportions was conducted using arcsine variance weights for fixed-effects and DerSimonian–Laird weights for random-effects models. For assessing associations, a log odds ratio was utilized to evaluate the relationship between associated factors and IPIs among food handlers in the included studies.

### 2.8. Research Questions

The framework for this systematic review and meta-analysis is guided by the following research questions: (1) What is the overall prevalence of intestinal parasites among university students and university food handlers in Ethiopia? (2) What factors can increase the prevalence of intestinal parasites among university students and university food handlers in Ethiopia?

## 3. Results

### 3.1. Study Selection

Out of a total of 101 identified published and unpublished studies, 45 duplicates were removed, leaving 56 unique studies. Among these, 35 studies were further excluded based on their titles, abstracts, being review articles, or not meeting the study's objectives. Out of the remaining 21 studies, six articles were excluded due to the unavailability of their full texts. Ultimately, 15 studies that met the inclusion criteria were included in the final meta-analysis (Figure 1).

### 3.2. Characteristics of Original Studies

Among the 15 studies conducted in Ethiopia between 2014 and 2024, a total of 101 study participants were involved in determining the combined prevalence of IPIs among university cafeteria workers and students. The majority of these studies employed a cross-sectional study design, with sample sizes ranging from 94 to 13,679 participants. Studies carried out at Bahir Dar University cafeteria (12.9%) [26], Wollo University cafeteria (15%) [18], and Woldia University cafeteria workers (16.8%) [27] reported the lowest prevalence of IPIs among food handlers. On the other hand, the highest prevalence was observed in a study at Wolkite University cafeteria (57.6%) [28], followed by Hawassa University cafeteria workers (47.9%) [21] and University of Gondar cafeteria workers (45.6%) [29].

Geographically, six studies were from the Southern Nations, Nationalities, and Peoples' Region (SNNPR) [21, 28, 30–33], five from the Amhara region [18, 26, 27, 29, 34], three from the Oromia region [20, 32, 35], and one from the Harar region [36]. Notably, there were no studies reported from the Benishangul Gumuz, Tigray, Gambela regions, or Dire Dawa. Regarding the quality assessment, the original studies' quality scores ranged from 2.5 to 4 (Table 1).

### 3.3. Pooled Prevalence of IPIs Among University Students and University Food Handlers in Ethiopia

**Figure 2:**
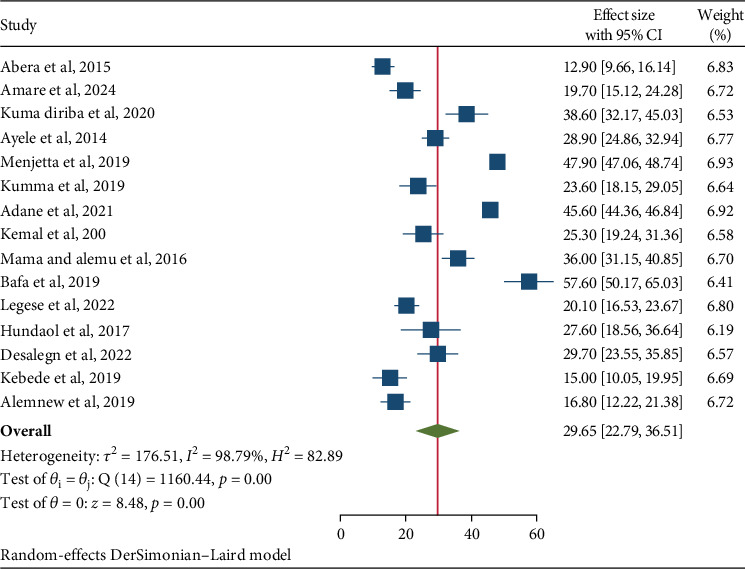
Forest plot of the pooled prevalence intestinal parasitic infections among university food handlers and university students in Ethiopia, 2024.

The prevalence of IPIs among food handlers in higher public university cafeterias and university students in Ethiopia was determined through the analysis of the 15 included studies, presented in a forest plot. The analysis revealed an overall prevalence of 29.65% (95% CI: 22.79, 36.5) (Figure 2). The analysis also indicated a high level of heterogeneity among the studies, with an *I*^2^ value of 98.79% and a *p* value less than 0.001. Consequently, a random-effects model was utilized to estimate the pooled prevalence of IPIs among food handlers in Ethiopia.

### 3.4. Specific Species Pooled Prevalence of Intestinal Parasite Infection

A high pooled prevalence of intestinal parasites was identified in mixed infections, specifically *E. histolytica/dispar* and *G. lamblia*, *A. lumbricoides* combined with *E. histolytica/dispar*, and *A. lumbricoides* along with hookworm. The pooled prevalence rates for these infections were 28.78% (95% CI: 24.33, 33.63), 19.27% (95% CI: 15.47–23.09), and 11.95% (95% CI: 8.81, 15.09), respectively. Furthermore, the pooled prevalence for specific species was as follows: *E. histolytica/dispar*: 11.52% (95% CI: 7.06, 15.98), *A. lumbricoides*: 9.34% (95% CI: 6.58, 12.1), *G. lamblia*: 5.85% (95% CI: 4.76, 6.95), hookworm: 2.29% (95% CI: 1.49, 3.096), and *Taenia* species: 1.66% (95% CI: 1.26, 2.06). Mixed infection of *A. lumbricoides*, *E. histolytica*, and *G. lamblia*: 1.24% (95% CI: 0.36, 2.12), *Hymenolepis nana*: 0.86% (95% CI: 0.67, 1.05), *Strongyloides stercoralis*: 0.29% (95% CI: 0.07, 0.51), *T. trichiura*: 0.24% (95% CI: 0.11, 0.36), *Enterobius vermicularis*: 0.22% (95% CI: 0.11, 0.33), *Schistosoma mansoni*: 0.50% (95% CI: 0.23, 0.77) among food handlers in Ethiopia. Notably, *E. histolytica/dispar* and *A. lumbricoides* were identified as the most prevalent intestinal parasites (Table 2).

### 3.5. Heterogeneity and Publication Bias

**Figure 3:**
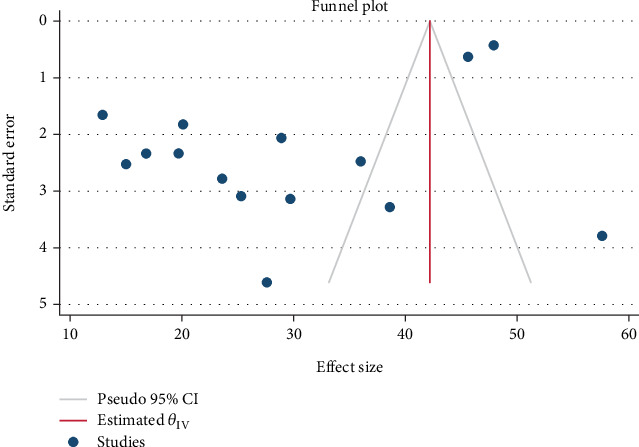
Funnel plot for the pooled prevalence intestinal parasitic infections among university food handlers and university students representing the evidence of publication bias in Ethiopia, 2024.

**Figure 4:**
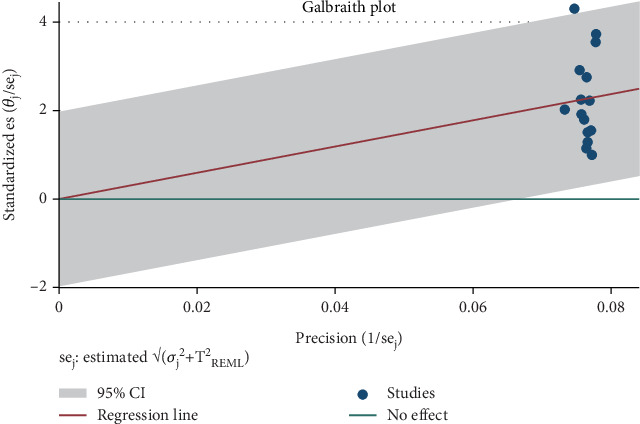
Egger's test for pooled prevalence intestinal parasitic infections among university food handlers and university students indicated the existence of publication bias among the included studies in Ethiopia, 2024.

The examination of the included studies revealed the presence of both heterogeneity and publication bias. Significant heterogeneity was observed across the 15 studies included in the meta-analysis, with an *I*^2^ value of 98.79% and a *p* value less than 0.001. The asymmetrical distribution seen in the funnel plot (Figure 3) may be linked to this identified heterogeneity or random variation. To determine if the observed asymmetry exceeded chance expectations, Begg's rank correlation test and Egger's regression intercept test were employed (Figure 4). The assessment of publication bias using Begg's and Egger's tests did not show any statistically significant bias in estimating the prevalence of IPIs among food handlers (*p* = 0.7072). Nevertheless, the presence of heterogeneity indicates the need for further investigation through subgroup analyses. Carrying out these analyses could be a prudent approach to pinpoint potential sources of heterogeneity and minimize the impact of publication bias on the overall prevalence estimates.

### 3.6. Subgroup Analysis

**Figure 5:**
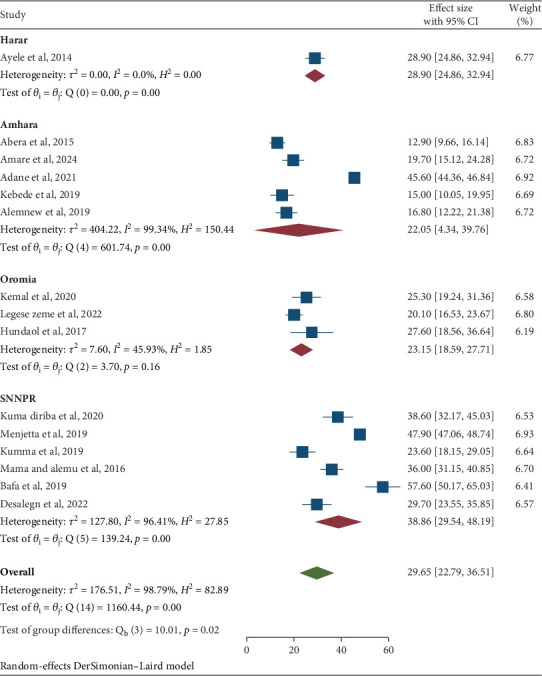
Subgroup analysis by region of the pooled prevalence of intestinal parasitic infections among university food handlers and university students in Ethiopia, 2024.

A comprehensive subgroup analysis was performed to evaluate the effects of sample size, geographical regions, study design, and laboratory detection methods on the pooled prevalence of IPIs among university students and food handlers. This analysis yielded significant insights and the results of the subgroup analysis revealed that the highest prevalence of IPIs was observed in the SNNPR, with a prevalence rate of 38.86% (95% CI: 29.54, 48.19). This was closely followed by the Harar region, which reported a prevalence of 28.90% (95% CI: 24.86, 33.94). Conversely, the Amhara region exhibited the lowest prevalence at 22.05% (95% CI: 4.34, 39.76), and the Oromia region had a prevalence of 23.15% (95% CI: 18.59, 27.71) (Table 3 and Figure 5).

Additionally, a subgroup analysis based on sample size indicated that studies with a sample size greater than 384 reported a combined prevalence of IPIs at 31.20% (95% CI: 20.80, 41.61). In contrast, studies with a sample size of less than 384 showed a prevalence of 28.80% (95% CI: 21.64, 35.96).

**Figure 6:**
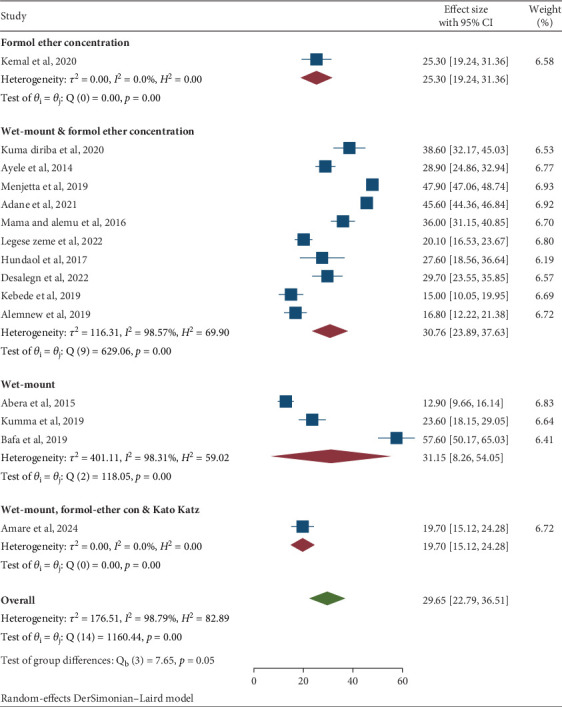
Subgroup analysis by laboratory detection methods of the pooled prevalence of intestinal parasitic infections among university students and university food handlers in Ethiopia, 2024.

Furthermore, an analysis focused on the laboratory methods used for detecting intestinal parasites revealed that the direct wet mount microscopy method had a relatively high detection rate for IPIs at 31.15% (95% CI: 8.26, 54.06) across three studies. This was significantly higher compared to the formol ether concentration method, which had a detection rate of only 5.30% (95% CI: 19.24, 31.36) in one study. When combining the wet mount and formol ether concentration methods, the overall detection rate was 30.76% (95% CI: 3.89, 37.63) across 10 studies. Moreover, the combination of wet mount, formol ether concentration, and Kato Katz techniques exhibited a detection rate of 19.70% (95% CI: 15.12, 24.28) in one study (Table 3 and Figure 6).

**Figure 7:**
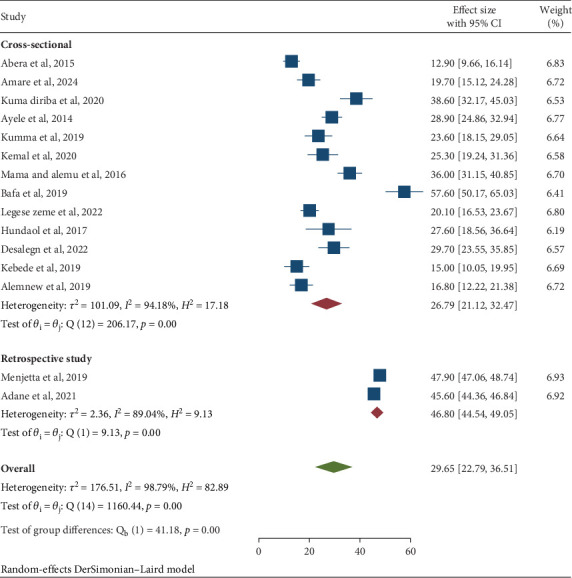
Subgroup analysis by study method of the pooled prevalence of intestinal parasitic infections among university students and university food handlers in Ethiopia, 2024.

Regarding the study design, retrospective studies reported the highest pooled prevalence of IPIs at 46.80% (95% CI: 44.54, 49.05), compared to cross-sectional studies, which showed a prevalence of 26.79% (95% CI: 21.12, 32.47) (Table 3 and Figure 7).

### 3.7. Sensitivity Analysis

**Figure 8:**
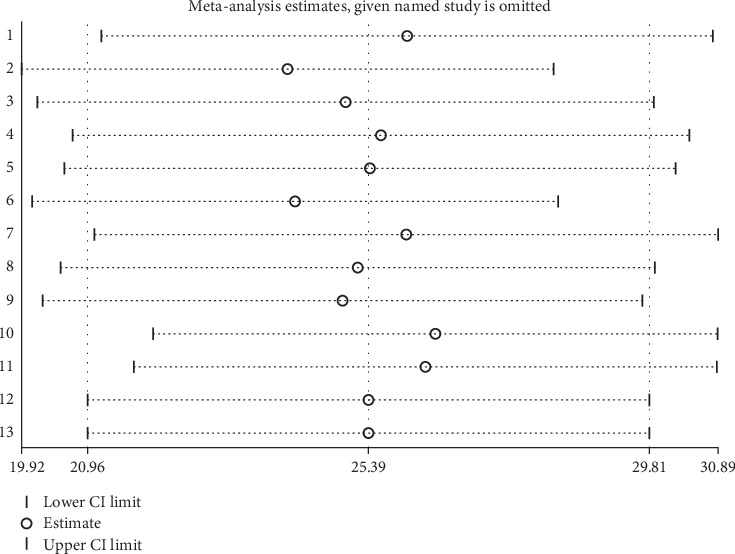
Sensitivity analysis results of the included studies that evaluated the influence of each study on the overall magnitude of intestinal parasite prevalence among university food handlers and university students in Ethiopia, 2024.

A sensitivity analysis was performed to examine the heterogeneity of the studies by systematically excluding one study at a time. This approach is aimed at determining whether the findings of each individual study had an impact on the overall prevalence of IPIs. This sensitivity analysis revealed that four studies (Abera et al., Menjetta et al., Adane et al., and Bafa et al.) significantly influenced the overall prevalence of IPIs among university students and food handlers in Ethiopia. Upon excluding these four studies, the recalculated prevalence estimate was found to be 25.39% (95% CI: 20.96–29.81) (Figure 8).

### 3.8. Factors Related to IPIs Among University Students and University Food Handlers in Ethiopia

**Figure 9:**
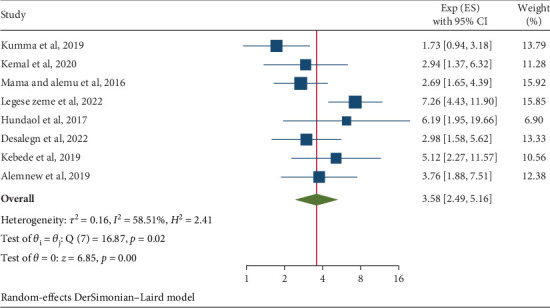
The pooled odds ratio of the association between intestinal parasitic infections and fingernail status among university food handlers and university students in Ethiopia, 2024.

**Figure 10:**
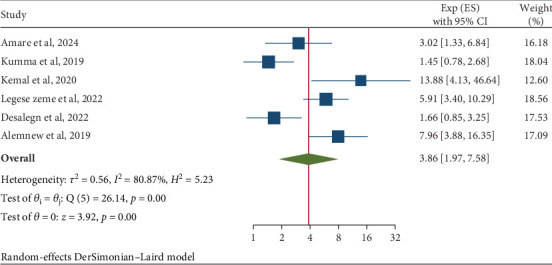
The pooled odds ratio of the association between intestinal parasitic infections and handwashing habits among food handlers in Ethiopia, 2024

This meta-analysis on IPIs among university students and food handlers in Ethiopia identified two significant risk factors associated with these infections. The factors identified were the condition of fingernails (OR = 3.58; 95% CI: 2.49, 5.16) and handwashing habits (OR = 3.86; 95% CI: 1.97, 7.58). Specifically, food handlers who did not regularly trim their nails had a 3.58 times higher likelihood of developing IPIs compared to those who maintained proper nail hygiene. The analysis also indicated a moderate level of heterogeneity (*I*^2^ = 58.51% and *p* = 0.02), which led to the application of a random-effects model (Figure 9). Furthermore, food handlers with inconsistent handwashing practices faced a 3.86 times increased risk of gastrointestinal parasites compared to individuals who practiced regular handwashing (Figure 10).

## 4. Discussion

IPIs are a major cause of morbidity and mortality among food handlers in Ethiopia [37]. A comprehensive assessment of the pooled prevalence of IPIs (IPIs) and their associated factors in Ethiopia can provide valuable insights for policymakers, enabling them to take corrective actions based on evidence. Therefore, this systematic review and meta-analysis was conducted to estimate the overall pooled prevalence of IPIs and the factors associated with these infections among food handlers in Ethiopia.

Furthermore, the findings offer a framework for implementing targeted and cost-effective control measures and centralizing and organizing this data could improve understanding and support the development of a comprehensive strategy for controlling IPIs. The systematic review and meta-analysis indicated an overall pooled prevalence of intestinal parasites among university students and food handlers in Ethiopia of 29.65% (95% CI: 22.79, 36.51).

This finding is consistent with previous systematic reviews and meta-analyses conducted in Ethiopia, which reported prevalence rates of 28.5%, 29.2%, and 33.6%, respectively [38–40]. Similarly, studies from southern Ethiopia documented a prevalence of 29.7% [41], while comparable rates were noted in Sudan (29.4%) [42], the Democratic Republic of Congo (28.6%) [43], and Spain (28%) [44]. In contrast, this prevalence rate is higher than the lower prevalence rates reported in Ethiopia by Tegen et al. (25.77%) [14], Harizanov et al. in Bulgaria (25.53%) [45], Staudacher et al. in Rwanda (25.4%) [46], and other studies from Iran [47], Sudan [48], Iran Southwest (8.8%) [4], Sari in Northern Iran (15.5%) [49], Shiraz (10.4%) [50], Thailand (10.3%) [51], and Egypt (26.5%) [52]. However, the pooled prevalence for intestinal parasites among food handlers in the present study is lower than findings from primary studies conducted in Venezuela (48.7%) [53], Brazil (47.1%) [54], Jordan (48.0%) [55], Turkey (59%) [56], Nigeria (54.8%) [57], and Rwanda (50.5%) [58].

The variations in prevalence rates can be attributed to differences in methodological approaches, sociodemographics, personal hygiene practices, and environmental factors between Ethiopia and other African countries. These factors may influence the prevalence of intestinal parasitic diseases, along with the impact of various elements on parasite transmission dynamics, such as social, cultural, and economic components, as well as the life cycles of the parasites.

The pooled prevalence of intestinal parasites among food handlers in the current study is lower than the findings from primary studies conducted in Venezuela (48.7%) [53], Brazil (47.1%) [54], Jordan (48.0%) [55], Turkey (59%) [56], Nigeria (54.8%) [57], and Rwanda (50.5%) [58]. The discrepancies in prevalence rates may be attributed to variations in methodological approaches, and various parasitological techniques are employed due to the absence of a gold standard test with 100% accuracy for detecting intestinal parasites. The prevalence estimate obtained through microscopy was lower (22.7%) compared to that achieved using molecular methods (61.4%), but slightly higher than when utilizing RDTs (14.5%). The disparities in laboratory techniques employed for diagnosing IPPs and the fluctuations in sensitivity and specificity, even within the same method, could potentially explain the differences in the observed IPPs rates in the current study. Moreover, advancements in clean water supply, health promotion strategies, and enhancements in personal and environmental hygiene may have played a role in decreasing the burden of intestinal parasites in Ethiopia.

The most commonly found intestinal parasites among university students and food handlers were *E. histolytica/dispar, G. lamblia*, *S. stercoralis*, *A. lumbricoides*, hookworms, *H. nana*, *Taenia* species, *E. vermicularis*, *S. mansoni*, and *T. trichiura*. Notably, *E. histolytica/dispar* had the highest prevalence at 11.52%, while *E. vermicularis* had the lowest at 0.22%. The pooled prevalence of amoebiasis among food handlers in this review aligns with a study conducted in Ethiopia, which reported a prevalence of 11% [39]. In contrast, the findings of the current study were lower than those from previous research conducted in Sudan (31.2%) by Suliman et al. [59], Côte d'Ivoire (56%) by Coulibaly et al. [60], Burkina Faso (66.5%) by Erismann et al. [61], and Iraq (88%) by Al-Taei [62].

Additionally, the prevalence found in Libya and Sari, Northern Iran, where the *E. histolytica/dispar* complex was 19.9% higher than the findings of this review [63]. However, the prevalence in this study was higher than that reported in earlier studies conducted by Walana et al. in Ghana (0.21%) [64], Rahi and Majeed in Iran (0.6%) [65], Sanprasert et al. in Thailand (0.73%) [66], Ismail KA in Egypt [67], Alemnew et al. [38] and Girma and Aemiro [40] from Ethiopia reported a prevalence of 6.38% and 6.78%, respectively.

The difference might be attributed to the aforementioned reasons, in addition to personal and cultural habits, which could result from poor hygiene practices given that the disease is transmitted through contaminated food, water, and fingers. The increasing trend could be linked to insufficient financial support, lack of political commitment, and inadequate community involvement in implementing effective strategies to reduce infection rates in Africa. The variations also may be differences in methodological approaches, sociodemographics, personal hygiene practices, and environmental factors between Ethiopia and other African countries. These factors may influence the prevalence of intestinal parasitic diseases, along with the impact of various elements on parasite transmission dynamics, such as social, cultural, and economic components, as well as the life cycles of the parasites.

The prevalence found in Libya and Sari, Northern Iran, where the *E. histolytica/dispar* complex was at 19.9%, was higher than the findings of this review [63]. However, the prevalence in this study was higher than that reported in earlier studies conducted by Walana et al. in Ghana (0.21%) [64], Rahi ang Majeed in Iran (0.6%) [65], Sanprasert et al. in Thailand (0.73%) [66], Ismail KA in Egypt [67], Alemnew et al. [38] and Girma and Aemiro [40] from Ethiopia reported a prevalence of 6.38% and 6.78%, respectively.

The disparities in prevalence rates may be related to the quality of food and water in different study locations, as well as their environmental conditions. Specifically*, E. histolytica/dispar* can contaminate drinking water and food, facilitating its spread through contaminated foods and vegetables. Additionally, the variation in prevalence rates between this review and other African countries might stem from differences in methodology, sociodemographics, personal hygiene practices, and environmental hygiene measures used to assess prevalence.

*A. lumbricoides* was the second most common intestinal parasite among food handlers, with a prevalence of 9.34%. The pooled prevalence of *A. lumbricoides* was lower in this study compared to national studies in Ethiopia [68] and sub-Saharan Africa [69], which may be attributed to variations in study populations, seasonal differences, sample sizes, methodologies, and diagnostic techniques. The relatively high prevalence of ascariasis in this study could be linked to inadequate personal hygiene, poor environmental sanitation, and the resilience of *Ascaris* eggs in harsh conditions. Moreover, the prolific egg production by female worms and the adhesive nature of *Ascaris* eggs contribute to their transmission through human hands, fruits, and vegetables.

In this meta-analysis, the pooled prevalence of *G. lamblia* was found to be 5.85% (95% CI: 4.76, 6.95). These results align with studies in Iran [4], Libya [70], Turkey [71], and Kenya [72], which reported prevalence rates of 4.52%, 4.9%, 6.1%, and 1.6%, respectively [49], and from Ethiopia and Alemenew et al. [38] and Tadesse et al. [68] reported a prevalence of 3.67% and 3.12%, respectively, Saudi Arabia (3%) [73], Cameroon (3.3%) [74], Iraq (4%) [65], and Thailand (4.2%) [66]. Furthermore, it is lower compared to earlier studies in various countries such as Ethiopia (10.03%) [14], Tanzania (10.6%) [73], Iraq (10.8%) [75], Ghana (12.2%) [64], and Nepal (12.5%) [63]. The discrepancy may be related to educational attainment, local sanitation practices, the availability of clean water, public health initiatives, and community awareness levels. Moreover, the climate and environmental conditions of an area can influence the parasite lifecycle and transmission patterns, resulting in regional disparities in prevalence rates. Additionally, differences in study methodologies, including the diagnostic techniques utilized, may contribute to the variability in reported prevalence rates. Certain diagnostic approaches with greater sensitivity and specificity can lead to more precise detection of parasitic infections.

Moreover, the prevalence was lower compared to earlier studies in various countries such as Ethiopia (10.03%) [14], Tanzania (10.6%) [73], Iraq (10.8%) [75], Ghana (12.2%) [64], and Nepal (12.5%) [63]. The discrepancies may be related to educational attainment, local sanitation practices, the availability of clean water, public health initiatives, and community awareness levels. Additionally, climate and environmental conditions can influence the life cycle and transmission patterns of parasites, resulting in regional disparities in prevalence rates. Differences in study methodologies, including diagnostic techniques, may also contribute to variability in reported prevalence rates, as certain methods with higher sensitivity and specificity can lead to more accurate detection of parasitic infections [76].

The pooled prevalence of hookworm was found to be 2.29%, aligning with similar studies in Ethiopia conducted by Aklilu et al. in Addis Ababa (2.1%) [11] and Sahlemariam and Mekete in Jimma (2.9%) [77]. The prevalence of this parasite may be attributed to poor personal hygiene and its straightforward mode of transmission, often originating from contaminated soil or surfaces tainted with feces. Moreover, differences in dietary practices, climatic conditions, and sociocultural factors between study locations and periods could explain the disparities observed in the research findings.

Additionally, this meta-analysis revealed the prevalence of *Taenia* species at 1.66% (95% CI: 1.26, 2.06), *T. trichiura* at 0.24% (95% CI: 0.11, 0.36), *H. nana* at 0.86% (95% CI: 0.67, 1.05), *E. vermicularis* at 0.22% (95% CI: 0.11, 0.33), *S. stercoralis* at 0.29% (95% CI: 0.07, 0.51), and *S. mansoni* at 0.50% (95% CI: 0.23, 0.77) among the common intestinal parasite infections. These findings are relatively consistent with studies in Southwest Iran, where *H. nana* was at 1.29%, and *E. vermicularis*, *T. trichiura*, and *S. stercoralis* were each reported at below 0.5% [69]. However, a study in Sari, Northern Iran, indicated *H. nana* at 1.9% as the only helminthic infection [49]. Moreover, a survey in Ethiopia found *A. lumbricoides* at 9.9%, hookworm at 9.7%, and *T. trichiura* at 2.6% to be the most common intestinal helminthes [63].

The prevalence of mixed IPIs among food handlers, particularly *E. histolytica* with *G. lamblia*, was significantly high at 28.78% (95% CI: 24.40, 33.16), while mixed infections of *A. lumbricoides*, *E. histolytica*, and *G. lamblia* were lower at 1.24% (95% CI: 0.36, 2.12). The high prevalence of mixed infections raises concerns about the complexity of treatment, as different parasites may require distinct therapeutic approaches. The coexistence of multiple parasites can exacerbate the severity of illness and lead to more adverse health outcomes, underscoring the critical need for prompt intervention.

Moreover, subgroup analysis revealed that the highest prevalence of IPIs was found in the SNNPR region at 38.78% (95% CI: 29.54, 48.19), followed by the Harar region at 28.90% (95% CI: 24.86, 32.94), and then Oromia at 23.15% (95% CI: 18.59, 27.71), while the Amhara region had the lowest prevalence at 22.05% (95% CI: 4.34, 39.76). The variation in prevalence among these regions may be attributed to differences in sociodemographic factors, environmental conditions, geographical characteristics, and behavioral practices, as well as local sanitation measures, the availability of clean water, public health initiatives, and community awareness regarding parasitic infections. Regions with inadequate sanitation and hygiene practices are more susceptible to higher rates of parasitic infections due to an increased risk of fecal–oral transmission.

This systematic review and meta-analysis is aimed at identifying factors associated with IPIs among food handlers in Ethiopia. The study revealed that practices such as fingernail trimming and handwashing habits were significantly associated with IPIs. Individuals who lacked handwashing practices were 3.86 times more likely to develop IPIs compared to those who practiced proper hand hygiene. This finding is consistent with studies from Kenya [78], Gambia [79], Pakistan [80], and previous research in Ethiopia [1, 31, 81], indicating an elevated risk of fecal–oral microorganism transmission. However, it was higher than findings from studies conducted in Ethiopia by [40], as well as in Nigeria by Amuta et al. [81], Indonesia by Pasaribu et al. [82], and Cameroon by Tchakounté et al. [83]. Effective handwashing practices may disrupt transmission routes for intestinal parasites.

The odds of acquiring IPIs were 3.58 times higher for individuals with untrimmed fingernails compared to those with trimmed nails. This result aligns with studies conducted in Ethiopia [14, 40, 84] and may be related to the accumulation of dust particles and parasites on untrimmed fingernails, facilitating fecal–oral transmission.

Furthermore, another subgroup analysis focused on the laboratory methods used for detecting intestinal parasite infections. Findings indicated that the direct wet mount microscopy method showed a relatively high detection rate for IPIs at 31.15% (95% CI: 8.26, 54.06) across three studies, compared to the formol ether concentration method, which had a detection rate of 5.30% (95% CI: 19.24, 31.36) in one study. Although the detection ability of wet mount for intestinal parasites is generally limited, its effectiveness was notably high in this review, likely due to the high prevalence of intestinal parasites in the study area.

This study does have limitations. All included studies were cross-sectional, which may have influenced the outcome variable due to confounding factors. Additionally, this systematic review and meta-analysis included research conducted over a range of time periods (2014–2024), which could impact prevalence rates and contribute to heterogeneity among studies. Furthermore, limited data and information from other parts of Ethiopia may restrict the conclusions drawn. Only a few published studies met the inclusion criteria and enhanced the current findings. Multiple parasitic diseases were unreported, and studies varied significantly across countries, with only English language publications considered.

## 5. Conclusions and Recommendations

The high prevalence of intestinal parasites among university students and food handlers in Ethiopia, particularly the notable rates of E*. histolytica/dispar* and *G. lamblia*, signifies a pressing public health concern that varies across geographic regions. The SNNPR university exhibits the highest prevalence, while the Amhara Region University shows the lowest. This geographic disparity necessitates a strategic adaptation of intestinal parasite control measures to the specific needs and characteristics of at-risk populations, as well as the local factors that contribute to parasite transmission. The studies referenced predominantly employed cross-sectional designs, revealing that larger sample sizes correlate with higher reported prevalence rates of IPIs. Furthermore, the effectiveness of different laboratory detection methods was highlighted, showcasing that direct wet mount microscopy is more reliable than the formol ether concentration method in identifying IPIs.

Moreover, personal hygiene practices among food handlers were identified as critical risk factors. Those who do not regularly trim their nails or practice routine handwashing are significantly more susceptible to IPIs. Specifically, the data indicate that these individuals are 3.58 times and 3.86 times more likely, respectively, to contract infections compared to their hygiene-compliant counterparts. In light of these findings, it is imperative for employers, managers, and owners of food and beverage establishments to take proactive measures to combat the spread of intestinal parasites. This includes implementing regular health screenings, conducting training sessions, and enforcing strict personal hygiene protocols for food handlers. The emphasis on routine parasite screenings and educational initiatives targeting proper nail trimming and hand hygiene practices is essential to prevent foodborne illnesses and protect the health of vulnerable populations. Therefore, the conclusion underscores the urgent need for tailored interventions that address the unique challenges posed by intestinal parasites in Ethiopia, highlighting the interplay of geography, study design, detection methods, and personal hygiene in the broader effort to reduce prevalence and enhance public health outcomes.

## Figures and Tables

**Table 1:** Characteristics of the 15 eligible studies of intestinal parasite infections included in systematic review and meta-analysis in Ethiopia, 2024.

**Ser no.**	**Authors**	**Pub_Year**	**Study design**	**Region**	**Participant**	**∗Lab methods**	**Place**	**Quality scores (5%)**	**Sample size**	**Prevalence (%)**
1	Abera et al.	2015	Cross-sectional	Amhara	Student cafteria workers	Wet mount	Bahir Dar University	3	410	12.9
2	Amare et al.	2024	Cross-sectional	Amhara	Student cafteria workers	Wet mount, formol ether con, and Kato Katz	University of Gondar	4	290	19.7
3	Kuma Diriba et al.	2020	Cross-sectional	SNNPR	Student cafteria workers	Wet mount and formol ether concentration	Dilla University	3	220	38.6
4	Ayele et al.	2014	Cross-sectional	Harar	Annual sport festival	Wet mount and formol ether concentration	Haramaya University	4	483	28.9
5	Menjetta et al.	2019	Retrospective study	SNNP	Student clinic	Wet mount and formol ether concentration	Hawassa University	2.5	13679	47.9
6	Kumma et al.	2019	Cross-sectional	∗SNNPR	Student cafteria workers	Wet mount	Wolaita Sodo University	3	233	23.6
7	Adane et al.	2021	Retrospective study	Amhara	Student clinic	Wet mount and formol ether concentration	University of Gondar	2.5	6244	45.6
8	Kemal et al.	2020	Cross-sectional	Oromia	Student cafteria workers	Formol ether concentration	MaddaWalabu University	3	198	25.3
9	Mama and Alemu et al.	2016	Cross-sectional	SNNPR	Student cafteria workers	Wet mount and formol ether concentration	ArbaMinch University	3	376	36
10	Bafa et al.	2019	Cross-sectional	SNNPR	Student cafteria workers	Wet mount	Wolkite University	3	170	57.6
11	Legese Zeme et al.	2022	Cross-sectional	Oromia	Student clinic	Wet mount and formol ether concentration	Adama University	4	483	20.1
12	Hundaol et al.	2017	Cross-sectional	Oromia	Student cafteria workers	Wet mount and formol ether concentration	Jimma University	3	94	27.6
13	Desalegn et al.	2022	Cross-sectional	SNNPR	Student cafteria workers	Wet mount and formol ether concentration	Wachemo University	3	212	29.7
14	Kebede et al.	2019	Cross-sectional	Amhara	Student cafteria workers	Wet mount and formol ether concentration	Wollo University	3	200	15
15	Alemnew et al.	2019	Cross-sectional	Amhara	Student cafteria workers	Wet mount and formol ether concentration	Woldia University		256	16.8

∗Lab, Laboratory; SNNP, South Nation Nationalities and Peoples Region.

**Table 2:** Specific species of pooled prevalence of intestinal parasites among university food handlers and university students in Ethiopia, 2024.

**Parasite**	**No study**	**Sample size**	**Positive**	**Pooled pre (95% CI)**	**I** ^2^ **(%)**	**Heterogeneity**	
						**Q**	**p**
*A. lumbercoides*	12	22,349	2700 (12.08%)	9.34 (6.58, 12.1)	98.55	828	< 0.001
*H. worm*	13	22,575	660 (2.90%)	2.29 (1.49, 3.10)	94.83	232.8	< 0.001
*T. trichuria*	9	21,569	59 (0.27%)	0.24 (0.11, 0.36)	69.25	26.02	< 0.001
*H. nana*	7	21,010	187 (0.89%)	0.86 (0.67, 1.05)	56.72	13.86	< 0.004
*E. vermicularies*	6	20,527	45 (0.219%)	0.22 (0.11, 0.33)	69.83	16.57	< 0.002
*S. stercolaries*	6	21,396	57 (0.266%)	0.29 (0.07, 0.51)	78.81	23.59	< 0.002
*Taenia* spp.	12	23,457	404 (1.72%)	1.66 (1.26, 2.06)	77.09	52.37	< 0.001
*S. mansoni*	4	20,333	102 (0.501%)	0.50 (0.23, 0.77)	93.13	43.67	< 0.001
*E. histolotica*	13	23,166	4074 (17.50%)	11.52 (7.06, 15.98)	99.40	2157.8	< 0.001
*G. lambila*	14	23,378	2011 (8.602%)	5.85 (4.76, 6.95)	91.20	159.7	< 0.001
*A. lum,E.h* and *G.l*	6	1429	27 (1.889)	1.24 (0.36, 2.12)	79.55	24.45	< 0.001
*E. hist* and *G. lam*	1	410	118 (28.7%)	28.78 (24.40, 33.16)	—	0.00	< 0.001
*A. lum* and *E. his*	1	410	79 (19.2%)	19.27 (15.47, 23,09)	—	0.00	< 0.001
*A. lum* and *H. worm*	1	410	49 (11.9%)	11.95 (8.81, 15.09)	—	0.00	< 0.001

Abbreviations: I, heterogeneity; Pre, prevalence; Q, Cochran's Q statistic.

**Table 3:** Subgroup analysis of the prevalence of IPIs among university cafeteria workers and university students.

**Variables**	**Characteristics**	**Included studies**	**Sample size**	**Pre (95% CI)**	**I** ^2^	**p** ** value**
Sample size	< 384	10	2249	28.80 (21.64, 35.96)	93.77%	*p* < 0.001
	> 384	5	21299	29.65 (22.79, 36.5)	99.40%	*p* < 0.001

Region	Amhara	5	11,090	22.05 (4.34, 39.76)	99.34	*p* < 0.001
	Oromia	3	775	23.15 (18.59, 27.71)	45.93	*p* > 0.05
	SNNPE	6	14890	38.86 (9.54, 48.19)	96.79	*p* < 0.001
	Harar	1	483	28.90 (24.86, 3.94)	0	*p* < 0.001

Study design	Cross-sectional	13	3625	26.79 (21.12, 3.47)	94.58	*p* < 0.001
	Retrospective	2	19923	46.80 (44.54, 49.05)	89.04	*p* < 0.001

∗Lab methods	Wet mount	3	813	31.15 (8.26, 54.06)	96.31	*p* < 0.001
	Formol ether concentration	1	376	5.30 (9.24, 31.36)	0	*p* < 0.001
	Wet mount and formol ether concentration	10	22,247	30.76 (3.89, 37.63)	98.57	*p* < 0.001
	Wet mount, formol ether concentration, and Kato Katz	1	290	19.70 (15.12, 24.28)	0	*p* < 0.01

∗I, heterogeneity; Lab, Laboratory; SNNPR, South Nation Nationalities and Peoples Region.

## Data Availability

The original data for this study are available from the corresponding author.

## Nomenclature

CI: confidence interval
IPs: intestinal parasites
OR: odds ratio
PRISMA: Preferred Reporting Items for Systematic Reviews and Meta-Analyses
SNNPR: South Nation Nationalities and Peoples Region
